# Design and Synthesis of Anti-MRSA Benzimidazolylbenzene-sulfonamides. QSAR Studies for Prediction of Antibacterial Activity

**DOI:** 10.3390/molecules16010175

**Published:** 2010-12-29

**Authors:** Marco Martín González-Chávez, Francisco Méndez, Roberto Martínez, Cuaúhtemoc Pérez-González, Fidel Martínez-Gutiérrez

**Affiliations:** 1 Programa de Doctorado en Ciencias Biológicas, Universidad Autónoma Metropolitana, México D.F., Mexico; 2 Facultad de Ciencias Químicas-CIEP, Universidad Autónoma de San Luis Potosí, San Luis Potosí, SLP, Mexico; 3 Departamento de Química, División de Ciencias Básicas e Ingeniería, Universidad Autónoma Metropolitana, Unidad Iztapalapa, México D.F., Mexico; 4 Instituto de Química, Universidad Nacional Autónoma de México, México D. F, Mexico; 5 Departamento de Sistemas Biológicos, Universidad Autónoma Metropolitana, Unidad Xochimilco, México D. F., Mexico

**Keywords:** benzimidazole, sulphonamide, MRSA, antibacterial activity

## Abstract

A series of benzimidazolylbenzenesulfonamide compounds containing electron-releasing and electron-withdrawing substituents were synthesized and tested for their *in vitro* antibacterial activity. Two BZS compounds showed strong antibacterial activity against methicillin-resistant *Staphylococcus aureus* and *Bacillus subtilis*. Quantitative studies of their structure-activity relationship using a simple linear regression analysis were applied to explore the correlation between the biological activity and the charges on acidic hydrogen atoms in the synthesized compounds.

## 1. Introduction

Drug resistance in Gram-positive bacteria is a worldwide public health problem with serious morbidity and mortality consequences. For example, the incidence of penicillin-resistant *Streptococcus pneumoniae* continues to increase worldwide, except in cities that apply good antibiotic use policies. An additional problem is the emergence of multi-drug resistant strains in America, Europe and Asia [1,2,3], for example, vancomycin resistance in *Enterococcus*. These infections have emerged as severe complications, mainly in critically ill patients in tertiary care hospital wards [4,5]. *Staphylococcus aureus* is one of the most prominent and widespread human pathogens, causing skin and tissue infections, deep abscess formation, pneumonia, endocarditis, osteomyelitis, toxic shock syndrome and bacteremia. Methicillin-resistant *Staphylococcus aureus* (MRSA) has been recognized as one of the main pathogenic causes of nosocomial infections throughout the world [6,7]. Infections caused by MRSA are a problem in health care institutions in the United States and worldwide, especially for intensive care unit patients [8]. Solving the problem of drug resistance will depend partially on the development of chemotherapeutic agents that selectively attack new bacterial targets.

Some benzimidazoles [9,11] and sulphonamides [12,13] have recently presented promising antimicrobial activities. Benzimidazolylbenzenesulfonamides show a variety of pharmacological activities due to their affinity toward various cellular protein targets or enzyme inhibitors, such as the peptide-like thrombin inhibitor [14], NR2B-selective *N*-methyl-D-aspartate antagonists [15], inhibitors of the enzyme 11*β*-hydroxysteroid dehydrogenate [16], and the human immunodeficiency virus type I integrase inhibitor [17]. 

**Figure 1:**
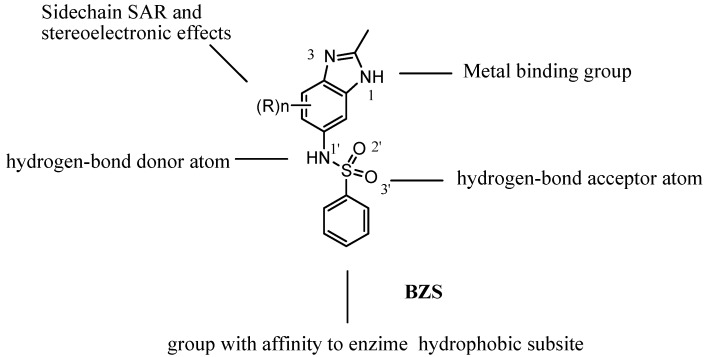
Design of BZS derivatives and the structure-activity relationships considered in this study.

Benzimidazolylbenzenesulfonamide (BZS) compounds fulfill the structural requirements for affinity toward metalloproteinases: a ligand group that may be directed toward a metal center, a sulfonamide group that may provide a hydrogen bonding interaction with the amino acids of the enzyme backbone, and an aryl group that may have effective Van der Waals interactions with enzyme subsites (Figure 1). Based on these considerations, we have focused on the synthesis of novel BZS compounds in search of synthetic chemotherapeutic agents. 

The present work establishes the regio-chemical, stereo-chemical, and electronic requirements of the benzimidazole ring of BZS for achieving activity toward Gram-positive bacteria, such as MRSA. The electronic requirements are modulated by the presence (or absence) of electron-releasing or electron-withdrawing substituents at positions 4, 5 and 7 of the benzimidazole ring of novel BZS derivatives.

### 1.1. Chemistry

**Scheme 1:**
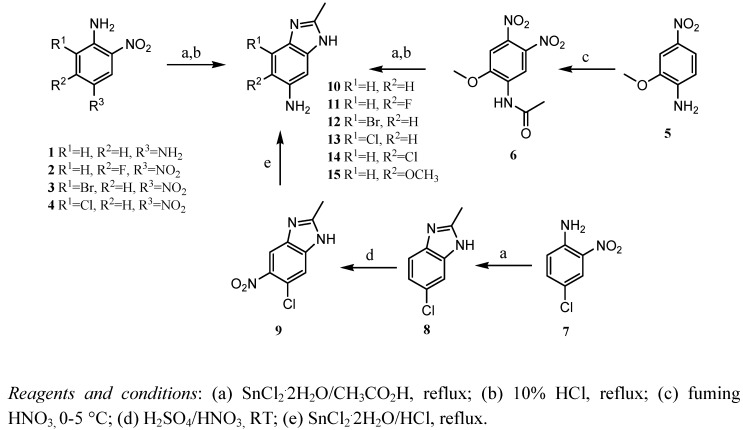
Synthesis of the benzimidazolamines **10**-**15**.

BZS compounds are usually obtained via benzimidazolamines**.** The synthesis of benzimidazol-amines **10-13** proceeded from the corresponding R_3_-dinitroaniline compounds **1-4**. The formation of the imidazole ring was carried out by reductive cyclization with acetic acid and tin (II) chloride dihydrate at reflux temperature, followed by acid hydrolysis in the presence of 10% hydrochloric acid at reflux temperature. The primary amine of benzimidazolamine **14** was obtained by nitration of benzimidazole **8** using a mixture of nitric and sulfuric acids, followed by reduction with tin (II) chloride dihydrate in hydro-chloric acid at reflux temperature. Synthesis of benzimidazolamine **15 **proceeded from the starting material nitroaniline **5**. The second nitro group necessary for formation of the imidazole ring was obtained by nitration of **5** with fuming nitric acid. Formation of the imidazole ring was done by reductive cyclization, similar to the procedure for formation of the imidazole ring in derivatives **10-13**. Finally, the benzimidazolamine **15** was obtained by acid hydrolysis of the amide group (Scheme 1).

**Scheme 2:**
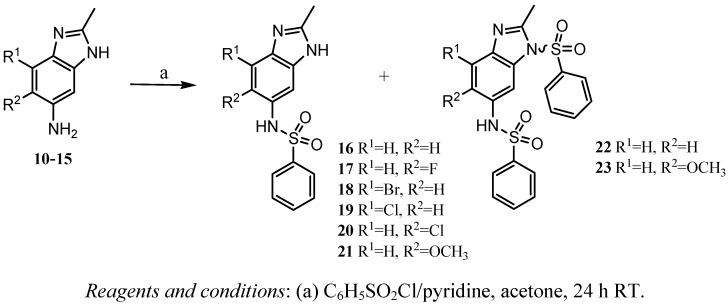
Synthesis of the BZS **16**-**23**.

The BZS **16-21** were obtained by reaction of benzimidazolamines **10-15** with benzenesulfonyl chloride in the presence of pyridine as a base and acetone as a solvent under N_2 _atmosphere, at room temperature for 24 h. Benzimidazolamines are bidentate nucleophiles, and produced the BZS derivatives **22 y 23 **(Scheme 2). This type of products was not obtained after of purification when the benzimidazole ring contained a halogen atom. 

**Scheme 3:**
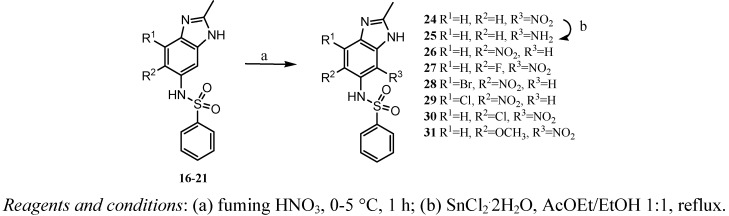
Synthesis of the BZSs **24**-**31**.

The derivatives of BZS that contained nitro groups, *i.e.*, **24** and **26-31**, were obtained from their **16-21 **precursors via nitration with fuming nitric acid. Only one mononitrated product was obtained in all cases, with the exception of derivative **16**. The BZS derivative containing an amino group were obtained by reduction of the nitro group of **24** using tin (II) chloride dihydrate in a 1:1 ethyl acetate/ethanol mixture (Scheme 3).

Compounds **10-26** were purified by column chromatography on silica gel using ethyl acetate/ hexane as the eluent. Compounds **27-31 **did not require chromatographic purification.

### 1.2. Microbiology

#### 1.2.1. Microbial strains and culture media

The Gram-positive microorganisms *Bacillus subtilis* (ATCC 6051), *Staphylococcus aureus* (ATCC 25923), methicillin-resistant *S. aureus* (MRSA) (ATCC 43300) and *Enterococcus fecalis* (ATCC 29212) were assayed. All microorganisms were grown in brain hear infusion (BHI) broth (BD, CA). Solid media was obtained by adding 1.5% (w/v) agar to the liquid media.

#### 1.2.2. Antibacterial activity

The antimicrobial activity of the synthesized compounds was determined on 96-well microdilution plates according to the protocols (published by the Clinical and Laboratory Standards Institute [18]) for determining the minimum inhibitory concentration (MIC) of each compound that results in inhibition of bacterial growth. Disposable micro titration plates were used for the antimicrobial tests. All compounds were dissolved in ethanol and diluted 2–512 times with 100 μL of Mueller–Hinton and bacteria were inoculated at a concentration of 10^5^ colony forming units (CFU mL^-1^). The MIC was measured after 20 h incubation at 37 °C with each substance tested. Growth inhibition of the bacterial strains was assessed by visual inspection. The antibacterial activities of the synthesized compounds were compared to oxacillin and ciprofloxacin as positive control. All assays were carried out in triplicate.

### 1.3. Theoretical calculations

Quantum chemical calculations of BZS **16**-**31** in the gas phase were performed using GAUSSIAN 03 [19] in conjunction with density functional theory (DFT). An extensive search for the lowest energy conformer on the potential energy surfaces of the BZS compounds was carried out at the B3LYP/3-21G(d) level, and geometry optimization of the lowest energy BZS conformer, followed by frequency calculations, was performed at the B3LYP/6-31G+(d,p) level. The atomic charges were obtained from a Mulliken population analysis. A simple linear regression analysis was performed using the StatGraphics Plus statistical software package.

## 2. Results and Discussion

All of the BZS compounds described above have not been previously reported. The structures of all synthesized compounds were confirmed by spectral data, and the IR, ^1^H-NMR, and mass spectra agreed with the proposed structures.

The synthesized compounds **16**-**31** were tested *in vitro* against four Gram-positive bacteria using a standard microdilution method and the control drugs (oxacillin and ciprofloxacin), as shown in Table 1 and Table 2. The results reported in Table 1 indicate that compounds **16**-**23** inhibited growth of the screened microorganisms, yielding MIC values in the range 125-500 μg/mL. However, the synthesized compounds exhibited low antibacterial potencies. 

**Table 1:** MIC (μg/mL) values for BZS compounds **16**-**23**.

Compounds	*S. aureus* ATCC 25923	MRSA ATCC 43300	*E. fecalis* ATCC 29212	*B. subtilis* ATCC 6633
**16**	>500	500	250	500
**17**	500	500	125	500
**18**	>500	500	250	>500
**19**	500	500	250	>500
**20**	500	500	125	500
**21**	>500	>500	250	500
**22**	500	500	250	375
**23**	>500	>500	500	>500

Table 2 reveals that the compounds **24**-**31** showed antibacterial activity against *S. aureus*, MRSA, *E. fecalis* and *B. subtilis* with MIC values in the range 2-500 μg/mL.

**Table 2:** MIC (μg/mL) values for BZS compounds **24**-**31**.

Compounds	*S. aureus* ATCC 25923	MRSA ATCC 43300	*E. fecalis* ATCC 29212	*B. subtilis* ATCC 6633
**24**	187	187	125	8
**25**	>500	>500	250	500
**26**	187	125	250	8
**27**	>500	500	500	86
**28**	16	10	125	2
**29**	31	18	125	2
**30**	500	500	500	86
**31**	>500	500	500	78
Oxacillin	0.25	2	8	>125
Ciprofloxacin	1 µg/mL	≤0.25 µg/mL	1 µg/mL	≤0.25 µg/mL

The structure-activity relationship (SAR) of compounds **16**-**31** was determined using the data presented in Table 1 and Table 2. SAR studies revealed that the presence of an electron-withdrawing group on the benzimidazole ring increased the antimicrobial activity, and activity decreased in the presence of electron-releasing atoms or groups. Specifically, compounds with a nitro group in either position 5 or position 7 of the benzimidazole core significantly increased potency against *S. aureus*, MRSA and *B. subtilis*. The dependence of compounds efficacy (biological activity) on the position of the nitro group did not appear to be important. The transformation of the nitro group in position 7 to an amino group led to loss of antibacterial activity. The substitution and region of a third atom or group in the nitro-BZS was decisive for increasing activity in MRSA and *B. subtilis*. The highest result antibacterial activity was exhibited by BZS containing a nitro group at position 5 and either chlorine or bromine atoms at position 4 of the benzimidazole ring. The importance of nitro group in enhancing the antimicrobial activity against Gram-positive bacteria is supported by similar results observed by Ören *et al.* [20] and Moro *et al.* [21] Exchange of the groups or atoms in the benzimidazole ring did not strongly inhibit the growth of *E. fecalis*.

The SAR study suggested that the acidity of hydrogens 1 and 1’ in the BZS molecule plays an important role in activity of against Gram-positive bacteria. Romero and Mendez showed that the charge on the hydrogen atom, calculated using DFT, is indicative of the gas phase acidity of substituted phenols, in agreement with the local hard and soft acids-bases principle (HSAB). [22] 

Based on this antecedent and with the purpose of quantifying a substituent’s effect on antibacterial activity, a study was carried out to investigate the quantitative structure-activity relationship (QSAR) by linear regression analysis of the –log MIC (μM) as a function of the charge on acidic hydrogen atoms and on BZS hydrogen bond acceptors.

The charge on hydrogen atoms (qH) that were hydrogen-bonded to the nitrogen atoms of BZS, as determined from the energetically favorable conformers of each BZS structure at the B3LYP/6-31+G(d,p) level, showed a larger charge for compounds containing Cl or Br atoms at position 4 and a nitro group at position 5 (Table 3) of the benzimidazole. These two compounds presented higher activities against MRSA and *B. subtilis*. The main differences between the conformations of BZS and with a NO_2_ group lay in the orientation of the NHSO_2_ hydrogen atom with respect to the NO_2_ group. 

**Table 3:** CMI and atomic charges on substituent atoms of BZS.

BZS	*B. subtilis* MIC (μM)	MRSA MIC (μM)	q H1	q N3	q H1’	q O2’	q O3’
**16**	1740	1740	0.304	-0.317	0.324	-0.533	-0.528
**17**	1639	1639	0.305	-0.315	0.334	-0.530	-0.532
**18**	-	1365	0.308	-0.267	0.326	-0.523	-0.530
**19**	-	1554	0.308	-0.302	0.325	-0.522	-0.529
**20**	1554	1554	0.305	-0.321	0.320	-0.516	-0.526
**21**	1575	-	0.301	-0.325	0.345	-0.531	-0.528
**24**	24	562	0.335	-0.321	0.382	-0.489	-0.467
**25**	1655	-	0.297	-0.346	0.348	-0.509	-0.559
**26**	24	376	0.308	-0.320	0.374	-0.470	-0.538
**27**	245	1427	0.333	-0.315	0.387	-0.494	-0.448
**28**	5	24	0.310	-0.232	0.365	-0.464	-0.479
**29**	5	49	0.312	-0.284	0.367	-0.479	-0.539
**30**	234	1363	0.335	-0.346	0.375	-0.421	-0.455
**31**	215	1380	0.332	-0.335	0.397	-0.453	-0.462

The linear regression analysis, which correlated the antibacterial activity with the atomic charges in the BZS derivatives containing a variety of substituents at positions 4, 5 and 7 of the benzimidazole ring (n = 12), showed that the best correlation between charge and antibacterial activity occurred between the qN3 and the –log MIC in *B. subtilis* and MRSA, with r > 0.6 (Table 4). 

**Table 4:** Correlation coefficients (r) for the linear regression analysis of atomic charges with antimicrobial activity of BZS derivatives. -log MIC = a + bq_k_.

Bacteria	N	q H1	q N3	q H1’	q O2’	q O3’
MIC *B. subtilis*	12^a^	0.332	0.651	0.596	0.591	0.270
	7^b^	0.916	0.747	0.888	0.956	0.337
	9^c^	0.889	-0.049	0.808	0.607	0.814
MIC MRSA	12^a^	-0.126	0.691	0.296	0.361	-0.039
	6^d^	0.938	0.882	0.798	0.868	0.530
	7^e^	0.579	-0.048	0.487	0.232	0.446

n = number of compounds. ^a^ BZS: **16**, **17**, **20**, **21**, **24**-**31**; ^b^ BZS: **16**, **17**, **20**, **21**, **26**, **28**, **29**; ^c^ BZS: **16**, **17**, **20**, **21**, **24**, **25**, **27**, **30**, **31** ; ^d^ BZS: **16**, **17**, **20**, **26**, **28**, **29**; ^e^ BZS: **16**, **17**, **20**, **24**, **27**, **30**, **31**.

In general, the parameters showed a high co-linearity with a correlation coefficient of r > 0.8 if the linear regression was performed by splitting the compounds into two groups: the first group comprised BZS with substituents in the 4 and 5 positions, and the second group comprised BZS with substituents in the 5 and 7 positions. This method yielded the best r values between qH1, qH1’, qO2’, and the –log MIC in *B. subtilis*, and between qH1, qN3, qO2’, and the –log MIC in MRSA, both for the BZS substituent positions 4 and 5. 

The best correlation models for the BZS derivatives that showed the greatest antibacterial activity are described in Equations 1-8 (Table 5) along with the statistical parameters for the linear regression analysis. The overall quality of the models was assessed by the correlation coefficient, *r*, the squared correlation, *r^2^*, the standard error, *S*, and the Fisher ratio, *F*.

**Table 5:** Linear regression analysis and quality of correlation for modeling the antimicrobial activity of BZS. –log MIC = a + bq_k._

Eq	QSAR Model (-log MIC=)	*n*	*r*	*r^2^*	*F*	*S*
	*B. subtilis*					
1	-94.5331 + 301.216 qH1	7	0.916	0.840	26.20	0.545
2	6.07449 + 27.504 qN3	7	0.747	0.558	6.31	0.905
3	-19.6851 + 50.298 qH1’	7	0.888	0.788	18.57	0.599
4	17.0515 + 38.3147 O2’	7	0.956	0.914	53.31	0.398
	MRSA					
5	-77.1726 + 242.811 qH1	6	0.938	0.880	29.40	0.321
6	3.63887 + 20.752 qN3	6	0.882	0.778	14.05	0.436
7	-12.128 + 27.5796 qH1’	6	0.798	0.637	7.02	0.559
8	8.95649 + 23.0719 qO2’	6	0.868	0.754	12.27	0.460

n = number of compounds.

The results suggested that the higher charges on H1 and H1’ in conjunction the lower charges on N3 and O2 promoted the activity of BZS derivatives against *B. subtilis* and MRSA.

## 3. Experimental

### 3.1. General

Melting points were determined using a Mel-Temp melting point apparatus in open capillary tubes and are uncorrected. IR spectra were collected on a Nexus 470 FT-IR spectrophotometer.^ 1^H-NMR spectra were recorded on Varian Gemini-200 MHz and Eclipse 300 MHz JEOL spectrometers in deuterated dimethyl sulfoxide (DMSO-d_6_) solutions using an internal TMS standard (0 ppm). The chemical shifts are reported in parts per million (δ/ppm). The peak patterns are indicated as follows: s, singlet; d, doublet; t, triplet; q, quartet; m, multiplet; br, broad. The coupling constants (*J*) are reported in Hertz (Hz). Mass spectra were detected by electronic impact (EI) and recorded using a JEOL JEM-AX505HA spectrometer by electronic impact (EI) of lower resolution at 70 eV. Flash column chromatography was carried out using silica gel 60 (230-400 mesh ASTM) from Merck.

### 3.2. General procedure for preparing benzimidazolamine *(10-13)*

A mixture of R_3_-nitroaniline **1**-**4** (13.06 mmol) and tin (II) chloride dihydrate (58.77 mmol for every nitro group) in acetic acid (20 mL) was heated under reflux for 4 h. The excess acetic acid was removed under reduced pressure, and the crude product was dissolved in 10% hydrochloric acid. The reaction mixture was heated under reflux for 1 h, was made alkaline by NaOH addition, and was extracted with ethyl acetate. The organic layer was dried over Na_2_SO_4_, and the solvent was evaporated under reduced pressure to given a crude product, which was purified by flash column chromatography on silica gel, using ethyl acetate:ethanol (90:10) as the eluent.

*2-Methyl-1H-benzimidazol-6-amine* (**10**). Yield: 84%; m.p. 86-88 °C. FT-IR (KBr, cm^-1^): 3399 (N-H), 3299 (N-H), 1639 (C=N, imidazole). ^1^H-NMR (200 MHz): δ 2.37 (s, 3H, CH_3_), 3.45 (s br, 2H, NH_2 _exchangeable with D_2_O), 6.43 (dd, 1H, Ar-H, *J* = 7.7 Hz), 6.59 (d, 1H, Ar-H, *J* = 1.8 Hz), 7.11 (d, 1H, Ar-H, *J* = 8.2 Hz). MS, m/z (Rel. Int.): 147 (M+,100), 119(8), 105 (12), 79 (13), 52 (13).

*5-Fluoro-2-methyl-1H-benzimidazol-6-amine* (**11**). Yield: 72%; m.p. 157-158 °C. FT-IR (KBr, cm^-1^): 3417 (N-H), 3371 (N-H), 1645 (C=N, imidazole). ^1^H-NMR (300 MHz): δ 2.36 (s, 3H, CH_3_), 4.69 (s br, 2H, NH_2_ exchangeable with D_2_O), 6.76 (d, 1H, Ar-H, *J* = 8.1 Hz), 7.08 (d, 1H, Ar-H, *J* = 11.4 Hz). MS, m/z (Rel. Int.): 165 (M+, 100), 137(5), 124(6), 97(9), 83 (7).

*4-Bromo-2-methyl-1H-benzimidazol-6-amine* (**12**). Yield: 66%; oil. FT-IR (KBr, cm^-1^): 3379 (N-H), 3338 (N-H), 1633 (C=N, imidazole). ^1^H-NMR (200 MHz): δ 2.38(s, 3H, CH_3_), 4.99 (s br, 2H, NH_2 _exchangeable with D_2_O), 6.54(d, 1H, Ar-H, *J* = 1.6 Hz), 6.65, (d, 1H, Ar-H, *J* = 1.8 Hz), 11.87 (s br, 1H, N-H exchangeable with D_2_O). MS, m/z (Rel. Int.): 225 (M+,100), 146 (19), 119 (9), 77 (10), 52 (7).

*4-Chloro-2-methyl-1H-benzimidazol-6-amine* (**13**). Yield: 69%; oil. FT-IR (KBr, cm^-1^): 3338 (N-H), 3391 (N-H), 1637 (C=N, imidazole). ^1^H-NMR (200 MHz): δ 2.38 (s, 3H, CH_3_), 5.07 (s br, 2H, NH_2 _exchangeable with D_2_O), 6.5 (s, 2H, Ar-H), 11.84 (s br, 1H, N-H exchangeable with D_2_O). MS, m/z (Rel. Int.): 181 (M+,100), 146 (13), 105(5), 78(11), 52 (5).

### 3.3. Procedure for preparing 5-chloro-2-methyl-1H-benzimidazol-6-amine (*14*)

To a suspension of 4-chloro-2-nitroaniline (4.7 mmol) in acetic acid (14.0 mL), was added tin (II) chloride dihydrate (0.021 mmol). The mixture was heated under reflux for 4 h. To the reaction solution was added NaHCO_3_ followed by extraction with ethyl acetate. The organic layers were dried over Na_2_SO_4_ and removed by evaporation under vacuum. The pure benzimidazole **8 **was obtained in 74% yield. 

To the benzimidazole **8** (1.8 mmol) was added a mixture of H_2_SO_4_/HNO_3_ (3:1, 1.2 mL). The reaction was stirred at room temperature for 1 h. To the mixture was added cold water. The resulting solid was filtered, washed with cold water, and dried under vacuum to afford pure benzimidazole **9** in 98% yield.

To a suspension of benzimidazole **9** (1.8 mmol) in HCl (5 mL) was added tin (II) chloride dihydrate (8.0 mmol). The mixture was heated under reflux for 4 h. The resulting solid was filtered, dissolved in aqueous 10% NaOH and the product extracted with ethyl acetate. The extract was dried and under vacuum to gave the desired benzimidazolamine **14**. Yield: 78%; m.p. 108 °C. FT-IR (KBr, cm^-1^): 3429 (N-H), 3354 (N-H), 1639 (C=N, imidazole). ^1^H-NMR (200 MHz): δ 2.40 (s, 3H, CH_3_), 4.94 (s br, 2H, NH_2_ exchangeable with D_2_O), 6.85 (s, 1H, Ar-H), 7.32 (s, 1H, Ar-H). MS, m/z (Rel. Int.): 181 (M+, 100), 146 (11), 105(4), 78(11), 52 (8).

### 3.4. Procedure for preparing 2-methyl-5-methoxy-1H-benzimidazol-6-amine (*15*)

To 2-methoxy-4-nitroaniline **5** (29.8 mmol) was added acetic anhydride (9.2 mL) and acetic acid (11.0 mL). The reaction was stirred under reflux for 2 h. To the mixture was added cold water, and the resulting solid was filtered, washed with cold water, and dried under vacuum. To this solid was added fuming HNO_3_ (12.0 mL), the reaction was stirred at 0 to 5 °C for 1 h. To the mixture was added cold water, and the resulting solid was filtered, washed with cold water, and dried under vacuum, to afford the pure acetamide **6** in 77% yield. From the acetamide **6,** the benzimidazolamine **15** was obtained following the general procedure for preparing benzimidazolamines **10**-**13**. Yield: 54%; oil. FT-IR (KBr, cm^-1^): 3417 (N-H), 3342 (N-H), 1637 (C=N, imidazole), 1140 (O-CH_3_). ^1^H-NMR (200 MHz): δ 2.36 (s, 3H, CH_3_), 3.76 (s, 3H, O-CH_3_), 4.4 (s br, 2H, NH_2_ exchangeable with D_2_O), 6.68 (s, 1H, Ar-H), 6.86 (s, 1H, Ar-H). MS, m/z (Rel. Int.): 177 (M+, 100), 162 (78), 134 (37), 89 (6), 52 (4).

### 3.5. General procedure for preparing benzimidazolylbenzenesulfonamides *16-23*

To a solution of benzimidazolamine (**10**-**15**, 6.8 mmol) and pyridine (6.8 mmol, 0.55 mL) in anhydrous acetone was added benzenesulfonyl chloride (6.8 mmol, 0.87 mL). The reaction mixture was stirred at room temperature under a N_2_ atmosphere for 24 h. Aqueous NaHCO_3_ was then added, and the mixture was extracted with ethyl acetate. The organic layer was dried over Na_2_SO_4_, filtered, concentrated, and purified by flash column chromatography on silica gel (gradient elution 50:50 hexane/ethyl acetate to 70:30 ethyl acetate/ethanol) to give **16**-**21** and **22**-**23**.

*N-(2-methyl-1H-benzimidazol-6-yl)benzenesulfonamide* (**16**). Yield: 68%; m.p. 221-222 °C. FT-IR (KBr, cm^-1^): 3302 (N-H), 1633 (C=N, imidazole), 1340 and 1155 (O_2_S-NH). ^1^H-NMR (300 MHz): δ 2.41 (s, 3H, CH_3_), 6.83 (d, 1H, Ar-H, *J* = 7.9 Hz), 7.14 (s, 1H, Ar-H), 7.5 (dd, 1H, Ar-H, *J* = 6 Hz), 7.54 (m, 3H, Ar-H), 7.68 (dd, 2H, Ar-H, *J* = 7.8 Hz). MS, m/z (Rel. Int.): 287 (M+, 36), 146 (100), 119 (7), 105 (4), 77 (5).

*N-(5-fluoro-2-methyl-1H-benzimidazol-6-yl)benzenesulfonamide* (**17**). Yield: 45%; m.p. 152 °C. FT-IR (KBr, cm^-1^): 3253 (N-H), 1635 (C=N, imidazole), 1323 and 1167 (O_2_S-NH). ^1^H-NMR (200 MHz): δ 2.43 (s, 3H, CH_3_), 7.17 (d, 1H, Ar-H, *J* = 10.2 Hz), 7.17 (d, 2H, Ar-H, *J* = 7.0 Hz), 7.57 (m, 5H, Ar-H), 9.91 (s br, 1H, NH exchangeable with D_2_O), 12.27 (s br, 1H, NH exchangeable with D_2_O). MS, m/z (Rel. Int.): 305 (M+, 18), 164 (100), 137 (12), 96 (7), 77 (11).

*N-(4-bromo-2-methyl-1H-benzimidazol-6-yl)benzenesulfonamide* (**18**). Yield: 66%; m.p. 272-273 °C. FT-IR (KBr, cm^-1^): 3290 (N-H), 1631 (C=N, imidazole), 1317 and 1163 (O_2_S-NH). ^1^H-NMR (200 MHz): δ 2.43 (s, 3H, CH_3_), 7.02 (d, 1H, Ar-H, *J* = 1.8 Hz), 7.15 (d, 1H, Ar-H, *J* = 2.0 Hz), 7.53 (m, 3H, Ar-H), 7.69 (dd, 2H, Ar-H, *J* = 8.2 Hz), 10.19 (s, 1H, NH exchangeable with D_2_O), 12.45 (s br, 1H, NH exchangeable with D_2_O). MS, m/z (Rel. Int.): 365 (M+, 27), 224 (100), 145 (14), 104 (5), 77 (17).

*N-(4-chloro-2-methyl-1H-benzimidazol-6-yl)benzenesulfonamide* (**19**). Yield: 57%; m.p. 358 °C. FT-IR (KBr, cm^-1^): 3284 (N-H), 1633 (C=N, imidazole), 1319 and 1162 (O_2_S-NH). ^1^H-NMR (200 MHz): δ 2.43 (s, 3H, CH_3_), 6.89 (d, 1H, Ar-H, *J* = 1.6 Hz), 7.11 (d, 1H, Ar-H, *J* = 1.8 Hz), 7.53 (m, 3H), 7.70 (dd, 2H, Ar-H, *J* = 7.9 Hz), 10.2 (s, 1H, NH exchangeable with D_2_O), 12.41 (s br, 1H, NH exchangeable with D_2_O). MS, m/z (Rel. Int.): 321 (M+, 26) 180 (100), 153 (6), 103 (2), 77 (8).

*N-(5-chloro-2-methyl-1H-benzimidazol-6-yl)benzenesulfonamide* (**20**). Yield: 53%; m.p. 224 °C. FT-IR (KBr, cm^-1^): 3275 (N-H), 1631(C=N, imidazole), 1292 and 1151 (O_2_S-NH). ^1^H-NMR (200 MHz): δ 2.45 (s, 3H, CH_3_), 7.20 (s, 1H, Ar-H), 7.46 (s, 1H, Ar-H), 7.6 (m, 5H, Ar-H), 9.82 (s br, 1H, NH exchangeable with D_2_O), 12.36 (s br, 1H, NH exchangeable with D_2_O). MS, m/z (Rel. Int.): 321 (M+, 20), 180 (100), 153 (7), 103 (2), 77 (7).

*N-(2-methyl-5-methoxi-1H-benzimidazol-6-yl)benzenesulfonamide* (**21**). Yield: 22%; m.p. 206-207 °C. FT-IR (KBr, cm^-1^): 3282 (N-H), 1633 (C=N, imidazole), 1325 and 1167 (O_2_S-NH). ^1^H-NMR (300 MHz): δ 2.42 (s, 3H, CH_3_), 3.38 (s, 3H, O-CH_3_), 6.86 (s, 1H, Ar-H), 7.26 (s, 1H, Ar-H), 7.47 (m, 2H, Ar-H, *J* = 7.6 Hz), 7.57 (m, 1H, Ar-H, *J* = 7.3 Hz), 7.61(dd, 2H, Ar-H, *J* = 7.8 Hz), 9.30 (s, 1H, NH exchangeable with D_2_O), 12.06 (s br, 1H, NH exchangeable with D_2_O). MS, m/z (Rel. Int.): 317 (36), 176 (100), 148 (32), 107 (6), 77 (7).

*N-(2-methyl-1-phenylsulfonyl-1H-benzimidazol-6-yl)benzenesulfonamide* (**22**). Yield: 2%; m.p. 146-148 °C. FT-IR (KBr, cm^-1^): 3427 (N-H), 1622 (C=N, imidazole), 1163 (O_2_S-NH). ^1^H-NMR (300 MHz): δ 2.76 (s, 3H, CH_3_), 7.23 (dd, *J* = 8.8 Hz), 7.14 (dd, *J* = 8.8 Hz), 7.07 (dd, 1H, *J* = 8.7 Hz), 10.12 (s, 1H, NH exchangeable with D_2_O). MS, m/z (Rel. Int.): 427 (M+, 52), 286 (100), 141 (41), 97 (6), 77 (45).

*N-(2-methyl-5-methoxi-1-phenylsolfonyl-1H-benzimidazol-6-yl)benzenesulfonamide* (**23**). Yield: 15%; m.p. 186-187 °C. FT-IR (KBr, cm^-1^): 3261 (N-H), 1601 (C=N, imidazole), and 1176 (O_2_S-NH), 1091 (O-CH3). ^1^H-NMR (200 MHz): δ 2.73 (s, 3H, CH_3_), 3.48 (s, 3H), 7.09 (s, 1H), 7.49 (m, 2H, *J* = 7 Hz), 7.63 (m, 5H), 7.78 (m, 1H, *J* = 7.6 Hz), 7.81 (s, 1H), 7.91 (dd, 2H, *J* = 8.2 Hz), 9.73 (s. 1H, NH exchangeable with D_2_O). MS, m/z (Rel. Int.): 457 (M+, 20), 316 (100), 175 (19), 141 (26), 77 (98).

### 3.6. General procedure for the synthesis of benzimidazolylbenzenesulfonamides *24, 26-31*

To benzimidazolylbenzenesulfonamide (**16**-**21**, 3.5 mmol) was added fuming HNO_3_ (2.0 mL). The reaction mixture was stirred at 0-5 °C for 1 h. Cool water was then added to provide a yellow solid. The solid was filtered, washed twice with water and dried. The mixture of **24** and **26** was purified by flash column chromatography on silica gel, using gradient elution, 75:25 to 55:45 hexane/ethyl acetate.

*N-(2-methyl-7-nitro-1H-benzimidazol-6-yl)benzenesulfonamide* (**24**). Yield: 76%; m.p. 182 °C. FT-IR (KBr, cm^-1^): 3410 (N-H), 3222 (N-H), 1630 (C=N, imidazole), 1493 and 1356 (NO_2_), 1311 and 1161 (O_2_S-NH). ^1^H-NMR (300 MHz): δ 2.71 (s, 3H, CH_3_), 7.19 (d, 1H, *J* = 8.7 Hz), 7.54 (dd, 2H, *J* = 8.1 Hz), 7.67 (m, 3H), 7.93 (d, 1H, *J* = 8.7 Hz), 10.47 (s br, 1H, NH exchangeable with D_2_O). MS, m/z (Rel. Int.): 332 (M+, 64), 191 (100), 131 (29), 104 (12), 77 (34).

*N-(2-methyl-5-nitro-1H-benzimidazol-6-yl)benzenesulfonamide* (**26**). Yield: 22%; m.p. 184 °C. FT-IR (KBr, cm^-1^): 3261 (N-H), 1643 (C=N, imidazole), 1464 and 1358 (NO_2_), 1307 and 1174 (O_2_S-NH). ^1^H-NMR (200 MHz): δ 2.49 (s, 3H, CH_3_), 7.13 (s, 1H), 7.58 (m, 5H), 8.05 (s, 1H), 10.04 (s br, 1H, NH exchangeable with D_2_O), 12.72 (s br, 1H, NH exchangeable with D_2_O). MS, m/z (Rel. Int.): 332 (M+, 58), 191 (100), 131 (26), 104 (6), 77 (59).

*N-(5-fluoro-2-methyl-7-nitro-1H-benzimidazol-6-yl)benzenesulfonamide* (**27**). Yield: 75%; m.p. 242 °C. FT-IR (KBr, cm^-1^): 3348 (N-H), 3265, 1639 (C=N, imidazole), 1522 (NO_2_), 1325 and 1167 (O_2_S-NH). ^1^H-NMR (200 MHz): δ 2.51 (s, 3H, CH_3_), 7.55 (m, 6H), 10.35 (s br, 1H, NH exchangeable with D_2_O), 12.95 (s br, 1H, NH exchangeable with D_2_O). MS, m/z (Rel. Int.): 350 (M+, 47), 209 (100), 192 (51), 122 (8), 77 (12).

*N-(4-bromo-2-methyl-5-nitro-1H-benzimidazol-6-yl)benzenesulfonamide* (**28**). Yield: 71%; m.p. 251 °C. FT-IR (KBr, cm^-1^): 3373 (N-H), 3215 (N-H), 1624 (C=N, imidazole), 1463 (NO_2_), 1309 and 1161 (O_2_S-NH). ^1^H-NMR (300 MHz): δ 2.53 (s, 3H, CH_3_), 7.13 (s, 1H), 7.54 (m, 2H), 7.64 (m, 3H), 10.33 (s br, 1H, NH exchangeable with D_2_O), 13.11 (s br, 1H, NH exchangeable with D_2_O). MS, m/z (Rel. Int.): 410 (M+, 92), 269 (82), 239 (21), 132 (14), 77 (34).

*N-(4-chloro-2-methyl-5-nitro-1H-benzimidazol-6-yl)benzenesulfonamide* (**29**). Yield: 92%; m.p. 140-141 °C. FT-IR (KBr, cm^-1^): 3394 (N-H), 3255 (N-H), 1624 (C=N, imidazole), 1369 (NO_2_), 1338 and 1172 (O_2_S-NH). ^1^H-NMR (300 MHz): δ 2.55 (s, 3H, CH_3_), 6.99 (s, 1H), 7.54 (m, 2H), 7.65 (m, 3H), 10.39 (s br, 1H, NH exchangeable with D_2_O). MS, m/z (Rel. Int.): 366 (M+, 100), 224 (97), 208 (33), 132 (12), 77 (37).

*N-(5-chloro-2-methyl-7-nitro-1H-benzimidazol-6-yl)benzenesulfonamide* (**30**). Yield: 67%; m.p. 277-278 °C. FT-IR (KBr, cm^-1^): 3379 (N-H), 3288 (N-H), 1631 (C=N, imidazole), 1520 (NO_2_), 1338 and 1163 (O_2_S-NH). ^1^H-NMR (300 MHz): δ 2.53 (s, 3H, CH_3_), 7.60 (m, 5H), 7.82 (s, 1H), 10.41 (s br, 1H, NH exchangeable with D_2_O), 13.04 (s br, 1H, NH exchangeable with D_2_O). MS, m/z (Rel. Int.): 366 (M+, 42), 224 (100), 208 (74), 132 (13), 77 (16). 

*N-(5-methoxy-2-methyl-7-nitro-1H-benzimidazol-6-yl)benzenesulfonamide* (**31**). Yield: 94%; m.p. 182 °C. FT-IR (KBr, cm^-1^): 3290 (N-H), 1622 (C=N, imidazole), 1544 (NO_2_), 1346 and 1140 (O_2_S-NH). ^1^H-NMR (300 MHz): δ 2.65 (s, 3H, CH_3_), 3.26 (s, 3H), 7.35 (s, 1H), 7.53 (m, 5H), 10.16 (s br, 1H, NH exchangeable with D_2_O). MS, m/z (Rel. Int.): 362 (M+, 58), 221 (100), 173 (78), 133 (3), 77 (11).

### 3.7. Procedure for preparing N-(7-amino-2-methyl-1H-benzimidazol-6-yl)benzenesulfonamide *(25)*

A mixture of benzimidazolilbenzenesulfonamide (**24**, 2.9 mmol) and tin (II) chloride II dihydrate (13.0 mmol) in ethyl acetate/ethanol 1:1 (100 mL) was heated under reflux for 4 h. The solvent was removed under reduced pressure and the crude product was added to aqueous NaHCO_3_.The mixture was subsequently extracted with ethyl acetate. The organic layer was dried over Na_2_SO_4_, filtered, and concentrated to give **25**. Yield: 65%; m.p. 225 °C. FT-IR (KBr, cm^-1^): 3471 (N-H), 3319 (N-H), 1628 (C=N, imidazole), 1311 and 1159 (O_2_S-NH). ^1^H-NMR (300 MHz): δ 2.39 (s, 3H, CH_3_), 4.89 (s br, 2H, NH_2 _exchangeable with D_2_O), 6.31 (d, Ar-H, *J* = 8.4 Hz), 6.43 (d, Ar-H, *J* = 8.4 Hz), 7.49 (m, 2H, Ar-H, *J* = 7.8 Hz), 7.59 (m, 1H, Ar-H, *J* = 7.5 Hz), 7.64 (dd, 2H, Ar-H, *J* = 7.6 Hz), 9.14 (s br, 1H, NH exchangeable with D_2_O), 11.91(s br, 1H, NHexchangeable with D_2_O). MS, m/z (Rel. Int.): 302 (M+, 9), 161 (100), 134 (9), 107 (4), 77 (14).

## 4. Conclusions

We have synthesized and evaluated the antibacterial activities of BZS analogs containing electron-releasing and electron-withdrawing substituents on the benzimidazole ring. SAR and theoretical studies of this series of compounds have shown that the antibacterial activity of BZS compounds may be related to the charges on the acidic hydrogen atoms in the BZS structure. Two compounds, **28** and **29**, have shown antibacterial activity against Gram-positive bacteria, which suggests that they may be members of a promising new class of BZS anti-MRSA agents. These compounds present a good starting point for modelling antimicrobial activity. Future work will focus on calculation of the absolute gas phase acidity of the hydrogens H1 and H1’ of BZS and a QSAR between the acidity of these hydrogens and antimicrobial activity.
